# A Case of Prolonged Bradycardia and Hypotension After Ingestion of Incorrect Guanfacine Formulation

**DOI:** 10.7759/cureus.77432

**Published:** 2025-01-14

**Authors:** John Coacci, Robert Schwaner

**Affiliations:** 1 Emergency Medicine, Stony Brook University Hospital, Stony Brook, USA; 2 Emergency Medicine and Medical Toxicology, Stony Brook University Hospital, Stony Brook, USA

**Keywords:** attention deficit hyperactivity disorder (adhd), clonidine, critical care, emergency medicine, guanfacine, ingestion, medical toxicology, medication error prevention, pediatric neurology, pediatric pharmacology

## Abstract

Guanfacine is an alpha-two agonist commonly manufactured in two formulations, an extended-release formulation primarily used for the treatment of attention-deficit hyperactivity disorder (ADHD) in the pediatric population, and an instant-release formulation primarily used for the treatment of hypertension in the adult population. Formulation errors can cause profound adverse effects, especially given the different intended uses for each patient population. We present the case of a six-year-old male who was mistakenly prescribed 2 mg of instant-release guanfacine as opposed to his usual 2 mg of extended-release guanfacine. He presented to the emergency department (ED) with lethargy, bradycardia, hypotension and eventually required initiation of vasopressors. He was admitted to the pediatric intensive care unit (PICU) due to hemodynamic instability, where he metabolized the ingestion and clinically improved over the subsequent 34 hours. This case was unique as it demonstrates a profound alteration in hemodynamics after a seemingly small medication error, which is not often reported for this medication. This case report also demonstrates the importance of recognizing specific medication formulations and highlights the need for careful medication prescribing and dispensing.

## Introduction

Guanfacine is an alpha-two agonist that is used for the treatment of various conditions in both the adult and pediatric populations. Two common formulations exist: an instant-release formulation used primarily for the treatment of hypertension, and an extended-release formulation used primarily for the treatment of attention-deficit hyperactivity disorder (ADHD) [1-3]. Extended-release guanfacine has gained popularity, particularly in the pediatric population, as it has been effective in reducing impulsivity and improving attention deficits while avoiding the adverse effects commonly associated with stimulant medications. Clinically significant overdoses are rarely reported, as the extended-release formulation has a more controlled release with much fewer fluctuations in concentration [4]. However, the pediatric population is often identified as a vulnerable population when considering the challenges associated with safe medication prescribing, dispensing, and administration. Relatively small changes in medication formulations can present profound clinical effects, attributed to the differences in pharmacokinetics and pharmacodynamics within this population [1,5,6]. We discuss the case of a six-year-old male who developed profound bradycardia and hypotension after he inadvertently received the same dose of instant-release guanfacine in place of extended-release guanfacine, requiring critical care and admission to the PICU until clinical resolution.

## Case presentation

A six-year-old male with a past medical history of ADHD presented to the pediatric emergency department (ED) under the care of his mother with the chief complaint of lethargy and somnolence. The patient’s mother stated that she was notified by the patient’s school nurse that he appeared fatigued and was behaving abnormally. Upon taking the patient home, he began having episodes of dry heaving, became increasingly pale, and thereafter started slumping over in his chair, prompting evaluation in the ED. The patient’s mother reports that he was only given one dose of guanfacine prior to going to school, as was typical for the patient’s daily medication regimen. After extensive investigation, a review of the patient’s external pharmacy records revealed that the patient had been inadvertently prescribed 2 mg of Tenex, an instant-release formulation of guanfacine instead of his usual 2 mg of Intuniv, an extended-release formulation of guanfacine.

**Figure 1:**
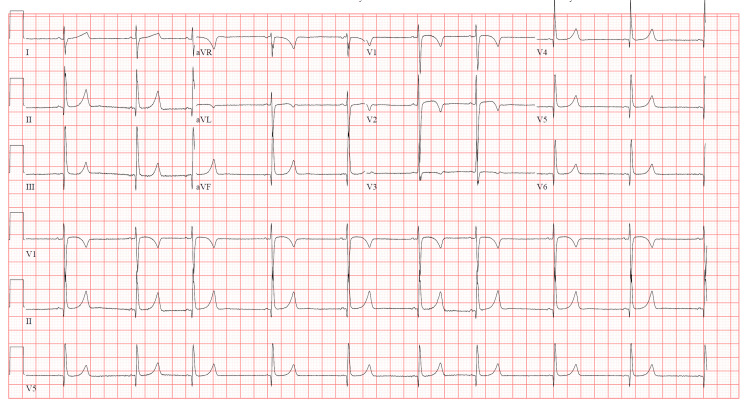
EKG completed approximately six hours post-ingestion.

Upon initial evaluation, the patient was pale, ill-appearing, and minimally responsive to verbal stimuli. His pupils were 4 mm, equal, and reactive bilaterally. Vital signs were significant for a heart rate of 69 and blood pressure of 69/48 mmHg. His oxygenation, EtCO2, and respiratory rate were acceptable. An electrocardiogram (EKG) revealed sinus bradycardia at 58, with a corrected QT interval of 404 milliseconds, and a QRS interval of 88 milliseconds (Figure 1). Chemistry and hematology studies, point-of-care glucose readings, and serum acetaminophen, salicylate, and ethanol levels were within normal limits. The patient received two peripheral intravenous (IV) lines and was administered one 20 cc/kg bolus of IV normal saline. Due to persistent symptomatic bradycardia and hypotension, he was administered an epinephrine infusion at 0.1 mcg/kg/min. His hemodynamics improved; however, he remained lethargic, only responding to occasional verbal stimuli. He was protecting his airway and did not require endotracheal intubation. He was admitted to the PICU for hemodynamic instability and intensive monitoring.

On hospital day (HD) 1, the epinephrine infusion was decreased to 0.08 mcg/kg/min (approximately 12 hours post-ingestion time). During an attempted wean to 0.06 mcg/kg/min the following morning (approximately 24 hours post-ingestion time), he had a recurrence of hypotension and bradycardia requiring up-titration of the epinephrine infusion, although his sedation was notably improved. Ultimately, he was weaned from the epinephrine drip completely during the subsequent 12 hours with improvement in his hemodynamics and clinical exam. The patient was discharged from the PICU in stable condition on HD 2, approximately 34 hours post-ingestion time, with no reported sequelae. Due to this profound reaction to the formulation change, the patient’s neurologist transitioned the patient off guanfacine completely.

## Discussion

Guanfacine is commonly prescribed for the treatment of ADHD in the pediatric population. It functions as a sympatholytic agent, sharing an imidazole structure with clonidine, a more familiar medication within the same pharmacologic class. Guanfacine primarily functions by stimulating alpha-two adrenergic receptors in the central nervous system, which reduces norepinephrine release from presynaptic neurons and ultimately decreases sympathetic outflow [1,2,7].

Guanfacine was originally developed as Tenex, an immediate-release formulation for the treatment of hypertension in the adult population. Later, an extended-release formulation manufactured as Intuniv was developed for the treatment of ADHD in pediatrics. The extended-release formulation has a longer duration of action, with peak serum concentrations occurring five to six hours post-administration, and a half-life ranging from 14 to 28 hours depending on the individual formulation [4,8]. This extended-release formulation also creates more stable serum concentrations with fewer fluctuations when compared to immediate-release [4].

Guanfacine has emerged as a popular non-stimulant option for the treatment of ADHD, due to its ability to improve attention and reduce impulsivity. The extended-release formulation specifically has been shown to reduce adverse effects, primarily somnolence, fatigue, and sedation, making it the preferred choice [4]. In addition to its use for ADHD, guanfacine is commonly prescribed off-label for conditions such as Tourette’s syndrome, anxiety, impulsivity, disruptive behaviors, and intrusive post-traumatic stress disorder (PTSD) symptoms [9].

Overdoses involving guanfacine are relatively uncommon, and like other pediatric ingestions, are often the result of exploratory behavior rather than intentional ingestion. Clinically significant overdoses are characterized by a sympatholytic toxidrome, which includes central nervous system depression, miosis, bradycardia, and occasionally hypotension [7]. Most overdoses are self-limiting, with symptoms resolving without residual effects [7,8]. Compared to clonidine, guanfacine has a greater affinity for alpha-2A adrenergic receptors, which theoretically results in a lower incidence of sedation and hypotension [2,3]. Nonetheless, lethargy and somnolence are often the predominant symptoms observed [1,3]. The development of clinically significant sympatholytic symptoms, particularly hypotension, may be delayed in patients who ingest extended-release formulations [9,10]. While peak hypotensive effects typically occur 12-18 hours after ingestion, there have been reports of orthostatic hypotension persisting for up to 48-60 hours post-ingestion [9,10]. This must be taken into consideration when caring for patients who ingest extended-release formulations of guanfacine.

The management of guanfacine overdose is generally supportive, with most cases resolving without the need for significant intervention. In most instances, bradycardia and mild hypotension can be managed with IV fluids and atropine if necessary [3]. Critical interventions, such as the use of vasopressors, inotropes, or endotracheal intubation are rarely required [1,7]. Despite the lack of a true specific antidote for guanfacine, the use of naloxone has been trialed. Although the exact mechanism of action is not known, it is postulated that naloxone blocks postsynaptic opioid receptors and opioid receptors in peripheral tissues, preventing inhibition of norepinephrine release and vasodilation [11]. However, its application remains controversial, and its efficacy remains inconsistent. Few case reports have suggested that naloxone may improve mental status and hemodynamics, potentially reducing the need for advanced interventions such as endotracheal intubation. Yet, these findings have not been consistently replicated in the literature [1,4,10,11]. Overall, the prognosis for guanfacine overdose is excellent, with most cases requiring only minimal intervention and resulting in low morbidity and mortality [7].

Medication errors are particularly concerning in the pediatric population. Pediatric patients may respond differently to medication toxicity due to variations in metabolism, weight-based dosing, and limited ability to report symptoms. Additionally, pediatric patients often compensate well which can further mask subtle signs of a clinically significant overdose or medication error. The risk of medication errors is further exacerbated by the complexities associated with pediatric dosing, compounding, and the involvement of multiple caregivers [1,4-6]. Children receiving medications such as guanfacine often have neurodevelopmental or behavioral disorders, which further increases the risk of dosing errors or overdoses [8]. This creates a high-risk environment that must be carefully managed when practicing safe medication prescribing, dosing, and administration involving both physicians and caregivers.

## Conclusions

Guanfacine is a medication primarily used for the treatment of ADHD in the pediatric population. The use of this medication has increased as it is considered a safe, non-stimulant alternative with overall successful control of impulsiveness and attention deficit. However, pediatric patients are especially sensitive to medication changes, and toxicity from this medication can produce varying degrees of sympatholytic toxidrome which warrants careful monitoring and may require intensive care. This case report demonstrates that seemingly simple formulation or dosing errors can necessitate prolonged critical care and further emphasizes the importance of safe medication prescribing and administration to prevent adverse outcomes.

## References

[1] Barbuto AF, Burns MM (2020). Clonidine compounding error: bradycardia and sedation in a pediatric patient. J Emerg Med.

[2] Fein DM, Hafeez ZF, Cavagnaro C (2013). An overdose of extended-release guanfacine. Pediatr Emerg Care.

[3] Minns AB, Clark RF, Schneir A (2010). Guanfacine overdose resulting in initial hypertension and subsequent delayed, persistent orthostatic hypotension. Clin Toxicol (Phila).

[4] Walton J, Byrum M, Shumaker A, Coury DL (2014). Prolonged bradycardia and hypotension following guanfacine extended release overdose. J Child Adolesc Psychopharmacol.

[5] D'Errico S, Zanon M, Radaelli D (2021). Medication errors in pediatrics: proposals to improve the quality and safety of care through clinical risk management. Front Med (Lausanne).

[6] Rickey L, Auger K, Britto MT (2023). Measurement of ambulatory medication errors in children: a scoping review. Pediatrics.

[7] Baumgartner K, Mullins M (2021). Pediatric clonidine and guanfacine poisoning: a single-center retrospective review. Toxicology Commun.

[8] Shimozato A, Ohashi K, Saitoh S (2023). Two case reports of extended-release guanfacine overdose in children. Pediatr Int.

[9] Mishra S, Shekunov J, Derscheid DJ, Canterbury EA, Leung JG (2022). Delayed signs and symptoms of extended release guanfacine overdose in two adolescent patients: implications of monitoring on the psychiatry unit. Case Rep Psychiatry.

[10] Spiller HA, Hays HL, Aleguas A Jr (2013). Overdose of drugs for attention-deficit hyperactivity disorder: clinical presentation, mechanisms of toxicity, and management. CNS Drugs.

[11] Tsze DS, Dayan PS (2012). Treatment of guanfacine toxicity with naloxone. Pediatr Emerg Care.

